# Noncanonical crRNAs derived from host transcripts enable multiplexable RNA detection by Cas9

**DOI:** 10.1126/science.abe7106

**Published:** 2021-04-27

**Authors:** Chunlei Jiao, Sahil Sharma, Gaurav Dugar, Natalia L. Peeck, Thorsten Bischler, Franziska Wimmer, Yanying Yu, Lars Barquist, Christoph Schoen, Oliver Kurzai, Cynthia M. Sharma, Chase L. Beisel

**Affiliations:** 1 Helmholtz Institute for RNA-based Infection Research (HIRI)/Helmholtz-Centre for Infection Research (HZI), 97080 Würzburg, Germany.; 2 Molecular Infection Biology II, Institute of Molecular Infection Biology, University of Würzburg. 97080 Würzburg, Germany.; 3 Core Unit Systems Medicine, University of Würzburg, 97080 Würzburg, Germany.; 4 Medical Faculty, University of Würzburg, 97080 Würzburg, Germany.; 5 Institute for Hygiene and Microbiology, University of Würzburg, 97080 Würzburg, Germany.; 6 Leibniz Institute for Natural Product Research and Infection Biology, Hans-Knoell-Institute, Jena, 07745 Germany.

## Abstract

CRISPR-Cas systems recognize foreign genetic material using CRISPR RNAs (crRNAs). In type II systems, a trans-activating crRNA (tracrRNA) hybridizes to crRNAs to drive their processing and utilization by Cas9. While analyzing Cas9-RNA complexes from *Campylobacter jejuni*, we discovered tracrRNA hybridizing to cellular RNAs, leading to formation of “noncanonical” crRNAs capable of guiding DNA targeting by Cas9. Our discovery inspired the engineering of reprogrammed tracrRNAs that link the presence of any RNA of interest to DNA targeting with different Cas9 orthologs. This capability became the basis for a multiplexable diagnostic platform termed LEOPARD (leveraging engineered tracrRNAs and on-target DNAs for parallel RNA detection). LEOPARD allowed simultaneous detection of RNAs from different viruses in one test and distinguished severe acute respiratory syndrome coronavirus 2 (SARS-CoV-2) and its D614G (Asp^614^→Gly) variant with single-base resolution in patient samples.

## Cellular RNAs guide CRISPR-Cas9

The Cas9 nuclease widely used for genome editing is derived from natural bacterial defense systems that protect against invading viruses. Cas9 is directed by RNA guides to cut matching viral DNA. Jiao *et al.* discovered that RNA guides can also originate from cellular RNAs unassociated with viral defense (see the Perspective by Abudayyeh and Gootenberg). They rendered this process programmable, linking the presence of virtually any RNA to cutting of matching DNA by Cas9. This capability is the basis of a new CRISPR diagnostic method developed by the authors that can detect many biomarkers at once. Named LEOPARD, this method can detect, for example, RNAs from severe acute respiratory syndrome coronavirus 2 and other viruses, thereby translating a new CRISPR discovery into a powerful diagnostic tool.

*Science*, abe7106, this issue p. 941; see also abi9335, p. 914

## Teaser

Cas9 guide RNAs can be derived from cellular RNAs, inspiring a multiplexable diagnostic platform.

CRISPR-Cas immune systems degrade foreign genetic material through the guidance of CRISPR RNAs (crRNAs) (*1*, *2*). crRNAs are encoded as spacer-repeat subunits within a system’s CRISPR array (*3*). Each crRNA typically undergoes processing from a precursor transcribed from the array and then partners with the system’s Cas effector nuclease to direct cleavage of target nucleic acids. Within type II systems, the source of Cas9 nucleases and many CRISPR technologies (*4*, *5*), crRNA processing and subsequent DNA targeting by Cas9 requires a trans-activating crRNA (tracrRNA) (*6*–*8*). The tracrRNA hybridizes to the “repeat” portion of each crRNA within the transcribed array. Host-derived ribonuclease (RNase) III then cleaves the formed RNA stem to generate a processed crRNA:tracrRNA duplex utilized by Cas9 (*6*). What remains unclear is whether crRNAs are confined to CRISPR-Cas loci or can be derived from elsewhere in the genome. Here, we show that crRNAs can be derived from host RNAs outside the CRISPR-Cas locus, inspiring a Cas9-based diagnostic platform that allows scalable detection of multiple biomarkers in a single test.

## Cellular RNAs bound to Cas9 from *C. jejuni* resemble crRNAs

**Fig. 1:**
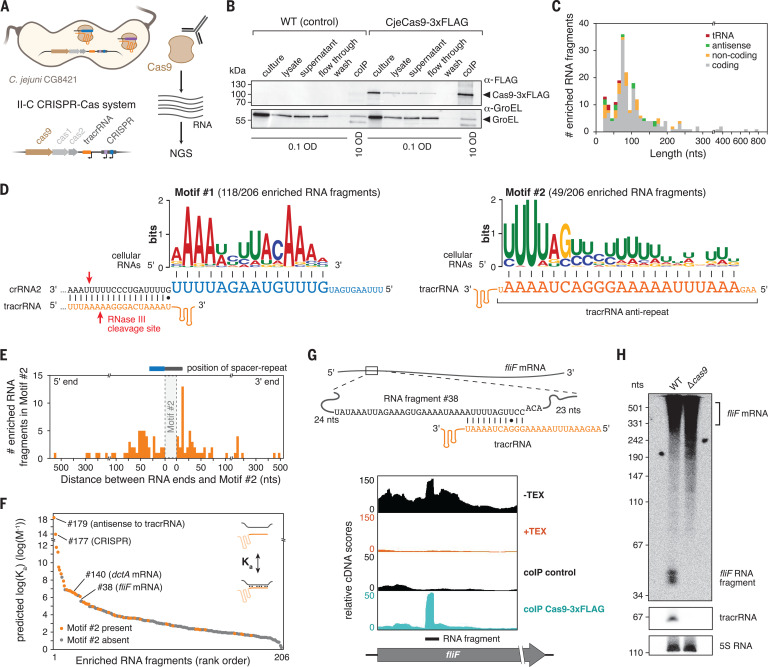
Fragments of cellular RNAs bound by Cas9 in *Campylobacter jejuni* resemble crRNAs. (**A**) Coimmunoprecipitation and sequencing RNAs bound to Cas9-3xFLAG from *C. jejuni* CG8421 using RIP-seq. (**B**) Western blot analysis of samples from *C. jejuni* strains with Cas9-3xFLAG or untagged WT control before and after immunoprecipitation. (**C**) Size range of the cellular RNA fragments identified through RIP-seq. Colors indicate the class of RNA. (**D**) Two motifs extracted by MEME from the enriched RNA fragments and their predicted interaction with crRNA2 or the tracrRNA. *E*-value = 8.8 × 10^−22^ (motif 1), 8.9 × 10^−14^ (motif 2). (**E**) Distribution of RNA lengths centered around motif #2. (**F**) Predicted binding affinity (*K*_a_) between the tracrRNA anti-repeat and each enriched RNA fragment. Orange indicates the presence of motif #2. (**G**) Predicted *fliF* mRNA:tracrRNA duplex and mapped reads from differential RNA-seq (top) or RIP-seq (bottom) performed in CG8421. (**H**) Northern blot analysis for *fliF* RNAs from CG8421 WT and *cas9*-deletion strains.

Our prior work interrogating RNAs bound to Cas9 from *Campylobacter jejuni* NCTC11168 (CjeCas9) revealed crRNA-guided RNA targeting by CjeCas9 (*9*). To further explore RNA binding partners of CjeCas9, we repeated the immunoprecipitation and RNA sequencing (RIP-seq) approach of epitope-tagged Cas9 using *C. jejuni* strain CG8421 harboring only two spacers in its endogenous type II-C CRISPR-Cas system (Fig. 1, A and B, and fig. S1, A and B). RIP-seq identified the CRISPR-tracrRNA locus as well as 205 RNA fragments derived from cellular RNAs enriched with Cas9-3xFLAG (Fig. 1C, fig. S1C, and table S1). Analyses of the enriched fragments using MEME (*10*) revealed two significant sequence motifs across all three replicates (Fig. 1D). Motif #1 was complementary to 13 nucleotides (nts) within the guide portion of crRNA2, in line with RNA targeting by crRNAs in NCTC11168 (*9*). Motif #2 was complementary to 21 nts within the tracrRNA anti-repeat domain. As this domain normally hybridizes to the crRNA repeat as part of crRNA biogenesis (fig. S2, A and B), motif #2 raised the possibility that these cellular RNAs were hybridizing with the tracrRNA, potentially becoming RNAs that function like crRNAs.

We explored this possibility through two routes. First, for the enriched RNA fragments with motif #2, we aligned the motif within each RNA fragment with a spacer-repeat pair, and we measured the length of each fragment corresponding to the spacer or repeat (Fig. 1E). Most frequently, the spacer part was 15 nt longer than a canonical crRNA spacer whereas the repeat part was the same size as a canonical crRNA repeat, similar to slightly extended versions of crRNAs. Second, we predicted how each RNA fragment base pairs with the tracrRNA anti-repeat (*11*). Predicted binding affinities were significantly higher for RNA fragments with motif #2 than for fragments without the motif (*p* = 3 × 10^−7^) (Fig. 1F). However, multiple RNA fragments were predicted to strongly pair with the tracrRNA anti-repeat despite lacking motif #2 (Fig. 1F), likely due to bulges in the RNA duplex creating discontinuities in the motif. For these RNA fragments and those containing motif #2, the predicted interactions between each RNA and the tracrRNA anti-repeat consistently contained imperfect RNA duplexes, with the most extensive pairing near the 3′ end of the anti-repeat (Fig. 1G and fig. S2). We made similar observations for motif #1 (fig. S1D).

These crRNA-like RNAs raised the question of whether these same RNAs were present in our prior RIP-seq analysis with strain NCTC11168 (*9*). We found that 7 of the 96 enriched fragments were predicted to bind the tracrRNA anti-repeat more tightly than at least one crRNA (fig. S3, A and B). Two of these RNAs (derived from *fliF* and *dctA* mRNAs) matched those found in CG8421 (Fig. 1G and figs. S2D and S3C). The RNA fragment derived from the *fliF* mRNA could be detected by Northern blot in both total RNA and RIP-seq samples yet disappeared following deletion of *cas9* (Fig. 1H and fig. S4, A and B). The *dctA* RNA fragment was only weakly detected in one strain (fig. S4). Although *cas9* deletion did not significantly perturb FliF protein concentrations in vivo under standard growth conditions (fig. S5), deleting the CRISPR array in NCTC11168 increased levels of the *fliF* RNA fragment (fig. S4C). Finally, the *fliF* RNA fragment was a processing product, as confirmed with Terminator exonuclease treatment (Fig. 1G). These crRNA-like RNAs thus are also present in *C. jejuni* NCTC11168 and likely exist in other *C. jejuni* strains on account of the shared tracrRNA binding site in *fliF* (fig. S6).

## Coimmunoprecipitated RNAs can function as noncanonical crRNAs that direct DNA targeting by Cas9

**Fig. 2:**
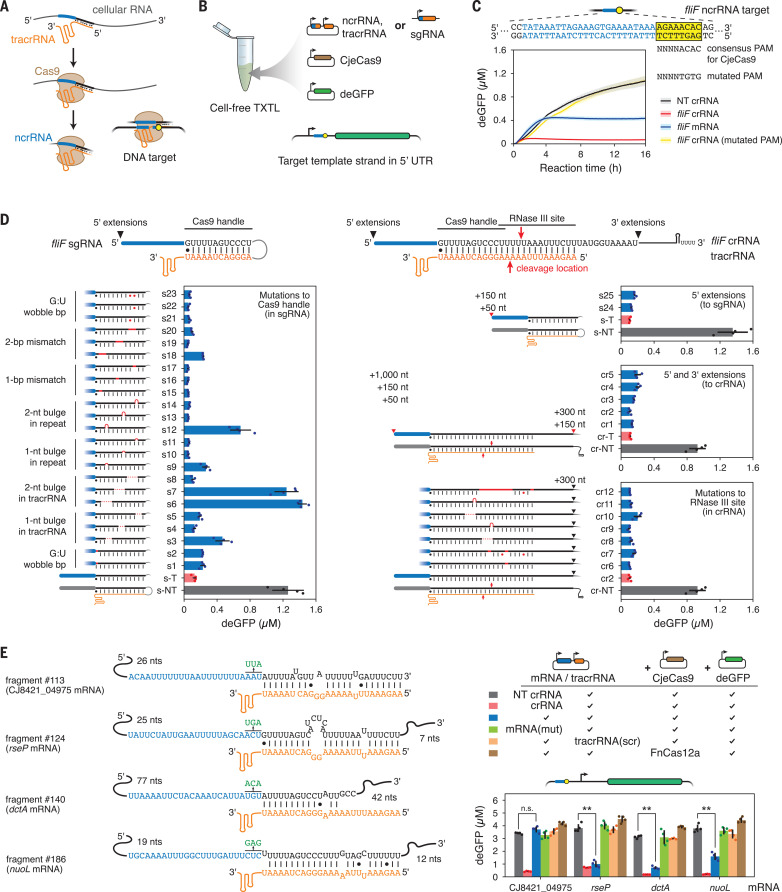
Noncanonical crRNAs can direct DNA cleavage by CjeCas9. (**A**) General process for ncrRNA generation. (**B**) Applying the TXTL assay to characterize putative ncrRNAs. (**C**) DNA targeting through the *fliF* ncrRNA in TXTL. Lines and shaded regions indicate the mean and standard deviation from four separately mixed replicates. NT, nontargeting. (**D**) Systematic evaluation of mutating the repeat:anti-repeat duplex for CjeCas9 with TXTL. Endpoint GFP levels are shown. Mutations and extensions to the *fliF* sgRNA-crRNA repeat are indicated in red. See table S1 for sequences. (**E**) DNA targeting by selected ncrRNAs predicted in TXTL. Check marks indicate use of the construct above the line. mRNA(mut): mRNA encoding the ncrRNA with point mutations in the predicted “seed” region of the guide. tracrRNA(scr): tracrRNA with the anti-repeat sequence scrambled. Values in (D) and (E) represent the mean and standard deviation from four separately mixed replicates. **p < 0.001. n.s., not significant.

Cas9 binding, predicted tracrRNA pairing, and the length distribution of many of these enriched RNA fragments suggested that the tracrRNA pairs with endogenous RNAs, resulting in “noncanonical” crRNAs (ncrRNAs) (Fig. 2A and fig. S2A). The ncrRNAs therefore would be expected to direct Cas9 to complementary DNA targets flanked by a protospacer-adjacent motif (PAM), similar to a canonical crRNA (*12*). As none of the genes giving rise to the detected ncrRNAs has a correctly placed PAM, the ncrRNAs are not expected to direct Cas9 to cleave their originating genomic site (table S1).

To evaluate ncrRNA-dependent targeting, we exploited a cell-free transcription-translation (TXTL) assay previously used to characterize CRISPR-Cas systems (*13*–*15*). As part of the assay, DNA constructs encoding CjeCas9, an RNA guide, and a green fluorescent protein (GFP) reporter harboring a target sequence flanked by a recognized PAM are added to the TXTL reaction. GFP fluorescence is then measured over time as a readout of DNA binding and cleavage by CjeCas9 (Fig. 2B and fig. S7A). We focused on examining the *fliF* ncrRNA given its presence in both *C. jejuni* strains and detection by Northern blotting. Applying this assay to the tracrRNA and mRNA comprising the entire *fliF* coding region (1683 nts) (Fig. 2C), we found that expressing the mRNA reduced GFP levels 2.5-fold compared with a nontargeting crRNA (*p* = 5.4 × 10^−5^). Expressing the equivalent crRNA reduced GFP levels 15.1-fold compared with the nontargeting control. The reduced GFP silencing for the *fliF* mRNA versus the crRNA potentially reflects not only reduced targeting efficiency but also delayed complex formation. Overall, the TXTL results offer evidence that mRNA-derived ncrRNAs can direct DNA targeting by Cas9.

The reduced performance of the *fliF* mRNA in TXTL could be due to how an ncrRNA deviates from a standard crRNA. These deviations include the crRNA repeat sequence, the secondary structure of the duplex formed with the tracrRNA anti-repeat, and 5′ or 3′ extensions to the repeat that do not undergo efficient processing. To evaluate these deviations, we systematically mutated or extended the standard crRNA, either as a single guide RNA (sgRNA) to ensure duplex formation or as a crRNA:tracrRNA pair, and evaluated GFP silencing in TXTL (Fig. 2D and table S1). CjeCas9 could accommodate some mutations within the region of the repeat:anti-repeat duplex in the sgRNA implicated in nuclease binding (*16*). The more disruptive mutations spanned more nts, were closer to the 5′ end of the repeat, or resulted in a bulge in the tracrRNA (e.g., s3, s6, s7, s9, s12, s18 in Fig. 2D, left). Observed differences in GFP silencing do not appear to arise from variable sgRNA levels (fig. S7B). Extending the sgRNA-crRNA ends or mutating the region cleaved by RNase III within the crRNA had minimal impact on GFP silencing (Fig. 2D, right). Overall, the tracrRNA can tolerate deviations from a standard crRNA as long as pairing through the 3′ end of the tracrRNA anti-repeat is maintained.

We applied insights from our mutational analyses to prioritize putative ncrRNAs from *C. jejuni* CG8421 for functional tests in TXTL. In total, we identified eight RNA fragments predicted to base pair extensively with the 3′ end of the tracrRNA anti-repeat (Fig. 2E and fig. S8). We then assessed GFP silencing by expressing up to 350 nts upstream and downstream of each associated ncrRNA-encoding gene with tracrRNA, CjeCas9, and the GFP reporter harboring each cognate DNA target. Of the eight tested RNAs, three (from *rseP*, *nuoL*, and *dctA*) yielded a >twofold reduction in GFP reporter levels compared with a nontargeting crRNA control (*p* < 0.001). Furthermore, targeting was directed specifically through the predicted ncrRNA, as mutating the “seed” region of the putative ncrRNA (*17*), scrambling the tracrRNA anti-repeat, or replacing CjeCas9 with the orthogonal FnCas12a nuclease fully relieved GFP repression (Fig. 2E). Multiple factors, such as mRNA folding or accessibility during translation, may explain why the other five ncrRNAs did not exhibit targeting activity in TXTL, as a linear-regression model built around the sgRNA mutants had limited ability to predict the targeting activity of these ncrRNAs (supplementary text S1). The current lack of predictability parallels guide design for RNA-targeting Cas13a nucleases, which only became predictable with extensive datasets and machine learning (*18*).

Beyond TXTL, we assessed ncrRNA function as part of DNA targeting in *C. jejuni* CG8421 and in *Escherichia coli*. For CG8421, transformation interference assays did not yield any significant DNA targeting directed by ncrRNAs derived from the *rseP*, *dctA*, and *nuoL* mRNAs (fig. S9A), likely due to low ncrRNA abundance compared with the strain’s crRNAs under the examined growth conditions. For *E. coli*, overexpressing the *dctA* mRNA, CjeCas9, and the tracrRNA led to moderate (15.5-fold) clearance of a transformed plasmid with the putative *dctA* ncrRNA target (*p* = 0.0036), but not when the tracrRNA anti-repeat was scrambled (1.6-fold) (*p* = 0.068) (fig. S9B). We therefore conclude that ncrRNAs derived from mRNAs can elicit DNA targeting in both in vivo and cell-free systems.

## The tracrRNA can be reprogrammed to direct Cas9 activity by an RNA of interest

**Fig. 3:**
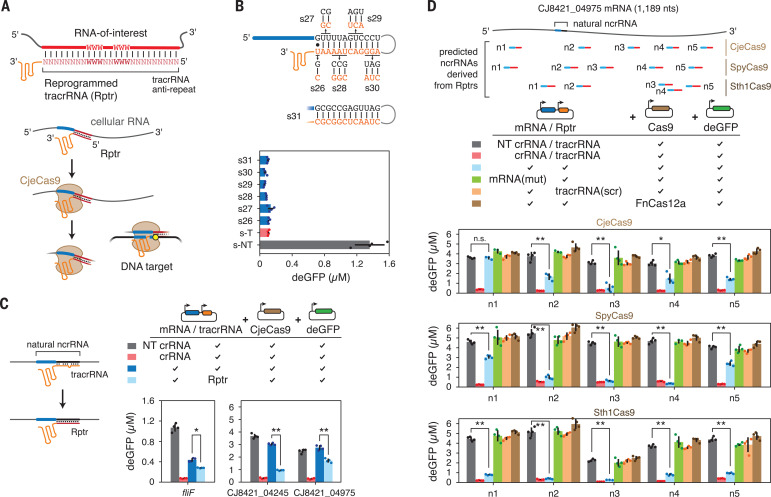
Reprogrammed tracrRNAs co-opt RNA transcripts to guide different Cas9 orthologs. (**A**) Design of reprogrammed tracrRNAs (Rptrs) utilized by CjeCas9. W = A or T. PAM, yellow circle. (**B**) Toleration of mutations to the RNA duplex of an sgRNA that preserve secondary structure in TXTL. (**C**) Enhancing DNA targeting by less functional or non-functional ncrRNAs by converting the tracrRNA into a Rptr in TXTL. (**D**) Sequence-specific DNA targeting in TXTL using Rptrs compatible with three different Cas9 nucleases. Values in (B) to (D) represent the mean and standard deviation from four replicates in TXTL. *p < 0.01; **p < 0.001. n.s., not significant.

The conversion of a cellular RNA into an ncrRNA was based on sequences bearing complementarity to the tracrRNA anti-repeat, analogous to natural crRNA biogenesis (fig. S2A). What if the tracrRNA anti-repeat sequence could be changed to hybridize to other RNAs while maintaining the appropriate structure for Cas9 recognition? If so, then the resulting reprogrammed tracrRNA (Rptr, pronounced “raptor”) could specifically derive an ncrRNA from a cellular RNA. The resulting ncrRNAs can then guide Cas9 to matching DNA targets (Fig. 3A). Although tracrRNA engineering has rarely been explored outside of sgRNAs or crRNA:tracrRNA duplexes (*19*), multiple studies have shown that the repeat:anti-repeat duplex of the sgRNA for the *Streptococcus pyogenes* Cas9 (SpyCas9) can be extensively modified as long as the secondary structure is maintained (*20*, *21*).

CjeCas9 recognizes a perfect RNA duplex formed between the crRNA repeat and tracrRNA anti-repeat (fig. S2B) (*16*). Based on our mutational analysis of the crRNA repeat, we already observed that Cas9 can accommodate several mutations within the crRNA repeat:tracrRNA anti-repeat duplex (Fig. 2D). Therefore, we evaluated GFP silencing in TXTL after mutating both sides of the duplex in the *fliF* sgRNA while preserving the secondary structure (s26-s31, Fig. 3B). GFP silencing was maintained even when exchanging the sequence of the entire duplex (s31, Fig. 3B). Next, we reprogrammed the tracrRNA anti-repeat to form perfect 25–base pair duplexes with three putative ncrRNAs (derived from *fliF*, CJ8421_04245, CJ8421_04975) exhibiting at most modest GFP silencing in TXTL (Fig. 2, C and E, and fig. S8). In all three cases, GFP repression was significantly enhanced with Rptrs compared with wild-type (WT) tracrRNAs (*p* = 1 × 10^−6^ to 0.0011), even if repression was not as strong as with the canonical crRNA:tracrRNA pair (Fig. 3C). Finally, we reprogrammed the tracrRNA anti-repeat to base pair with entirely new regions of an mRNA (Fig. 3D). Starting with the CJ8421_04975 mRNA, we designed five different CjeCas9 Rptrs hybridizing to different locations (n1 to n5) in the mRNA (Fig. 3D and fig. S10A). Of these Rptrs, four yielded significantly reduced GFP levels compared with nontargeting crRNA controls (*p* = 6 × 10^−7^ to 0.002). Notably, mutating the predicted seed region, scrambling the tracrRNA anti-repeat, or replacing CjeCas9 with FnCas12a restored GFP expression (Fig. 3D). Northern blot analysis from TXTL-extracted RNAs further revealed no detectable processed RNAs with a size resembling that of mature ncrRNA. Complete ncrRNA processing to a size similar to that of canonical crRNAs therefore may not be necessary for DNA targeting by CjeCas9 (fig. S11), in line with the dispensability of RNase III for crRNA-mediated DNA targeting through the II-C CRISPR-Cas system in *Neisseria meningitidis* (*22*).

Given the functionality of CjeCas9 Rptrs, we asked whether tracrRNAs for other Cas9 homologs can be similarly reprogrammed. We selected the well-characterized *Streptococcus pyogenes* Cas9 (SpyCas9) and the *Streptococcus thermophilus* CRISPR1 Cas9 (Sth1Cas9) as examples. In both cases, we devised design rules for Rptrs based on the known secondary structure of the crRNA:tracrRNA duplex and the preference of RNase III to cleave double-stranded RNA with AT-rich sequences (fig. S10A) (*20*, *23*). All 10 designed Rptrs significantly reduced GFP levels compared with the nontargeting crRNA control (*p* = 1 × 10^−7^ to 1 × 10^−4^) (Fig. 3D). As before, GFP expression was restored by disrupting the seed sequence in the ncrRNA guide, scrambling the tracrRNA anti-repeat, or swapping either of the Cas9’s for FnCas12a. In many cases, the extent of GFP silencing approached that of the targeting crRNA control. We also evaluated plasmid clearance with Rptrs in *E. coli* for all three Cas9 orthologs, finding that each could elicit efficient plasmid clearance for at least one tested Rptr (fig. S10B). The targeted plasmid was efficiently cleared even when expressing the sensed mRNA at low levels (fig. S12A) or when deleting RNase III (fig. S12B). Overall, the tracrRNA for different Cas9 orthologs can be converted into Rptrs to elicit DNA targeting based on the presence of a selected cellular RNA.

## Reprogrammed tracrRNAs enable sequence-specific detection by Cas9

**Fig. 4:**
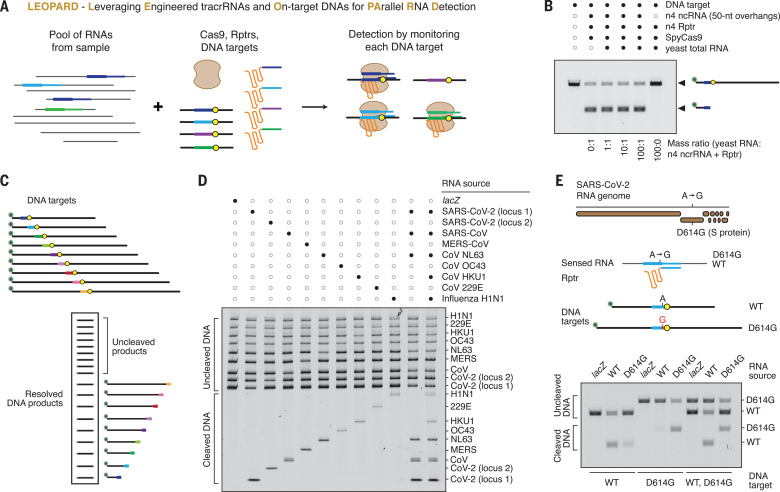
Reprogrammed tracrRNAs enable multiplexable RNA detection with single-base resolution in vitro. (**A**) Overview of the multiplexed diagnostic platform LEOPARD. (**B**) Highly specific cleavage of target DNA by SpyCas9 in vitro*.* Targeting was directed by the ncrRNA associated with the n4 locus in CJ8421_04975. (**C**) Multiplexed monitoring of DNA targets. (**D**) Parallel detection of nine different RNA fragments associated with respiratory viruses by denaturing polyacrylamide gel electrophoresis. Filled dots in (D) and (E) indicate the presence of the component listed on the right. (**E**) Specific detection of the D614G point mutation within the spike protein of SARS-CoV-2. Each detected T7-transcribed RNA in (B), (D), and (E) comprised the intended ncrRNA sequence with 50-nt extensions on either end.

By linking DNA targeting to an RNA of interest, Rptrs offer a valuable opportunity for RNA detection and a different paradigm for CRISPR diagnostics. Current CRISPR diagnostics principally rely on Cas12a or Cas13 searching for double-stranded DNA or RNA targets in a sample, where target recognition elicits nonspecific single-stranded DNA or RNA cleavage of a fluorescent reporter (*24*–*26*). The nonspecific readout practically limits one test to one target sequence. By contrast, Rptrs convert sensed RNAs into ncrRNAs, which would direct Cas9 to matching DNA. Cas9 binding or cleavage of a DNA sequence would then indicate the presence of the sensed RNA in the sample. Because the sequence of each DNA target is distinctive, large numbers of target sequences could be monitored in parallel in one test. We call the resulting diagnostic platform LEOPARD, for leveraging engineered tracrRNAs and on-target DNAs for parallel RNA detection (Fig. 4A).

To begin assessing LEOPARD, we performed a simplified in vitro reaction using T7-transcribed RNAs, commercially available SpyCas9 protein, and linear DNA targets (fig. S13A). We began with RNA corresponding to one of the synthetic ncrRNA loci within CJ8421_04975 (n4 under SpyCas9, Fig. 3D). Introducing an annealing step to hybridize the Rptr to the T7-transcribed ncrRNA yielded DNA target cleavage without adding RNase III or RNase A for ncrRNA:Rptr processing (fig. S13, B and C). The cleavage efficiency was also similar to that of the equivalent crRNA:tracrRNA pair, even when the ncrRNA sequence was extended on either end (fig. S13D). The time scale of the annealing step could also be minimized by rapid cooling of the samples (fig. S13E). With the annealing step, efficient cleavage occurred with a 100-fold excess of yeast total RNA but only when the ncrRNA was present (Fig. 4B). LEOPARD therefore can report the presence of a specific RNA of interest based on cleavage of a DNA target and can be streamlined through further optimization.

## LEOPARD allows for multiplexed RNA detection by Cas9 with single-base resolution

Realizing the full multiplexing potential of LEOPARD requires monitoring many DNA targets at once. To initially demonstrate this multiplex capability, we devised a readout scheme based on resolving distinct cleavage products from pooled DNA targets by gel electrophoresis (Fig. 4C). Each target is labeled with a fluorophore on one end, producing only two visualizable products—cleaved and uncleaved. We then applied this scheme to specifically detect nine ~150-nt RNA fragments associated with respiratory viruses, including two from SARS-CoV-2 coronavirus (the causative agent of COVID-19), six from other coronaviruses, and one from influenza H1N1 (Fig. 4D and fig. S14). Each DNA target was cleaved by Cas9 only in the presence of the corresponding RNA, even when detecting three or five specific RNA fragments in the same reaction (Fig. 4D and fig. S14).

As the viral RNA sequences were selected to minimize homology, we asked if LEOPARD could detect even a single-nucleotide difference. The Asp^614^→Gly (D614G) mutation in the spike protein of SARS-CoV-2 served as an example, as it comprises a single base change (A23403G) that increased infectivity and drove global spread (*27*). By placing this nt change within the seed region of the target, we could detect the WT or D614G RNA using one Rptr combined with either the WT or D614G target (Fig. 4E). The matching DNA target was preferentially cleaved when testing each target individually, although some cleavage of the nonmatching target was observed. However, combining the two targets in a single reaction yielded discernable cleavage only for the matching target, presumably through preferential binding and cleavage of the perfect target by Cas9 (*28*). LEOPARD therefore can confer multiplexed RNA detection in a single reaction with single-base resolution.

**Fig. 5:**
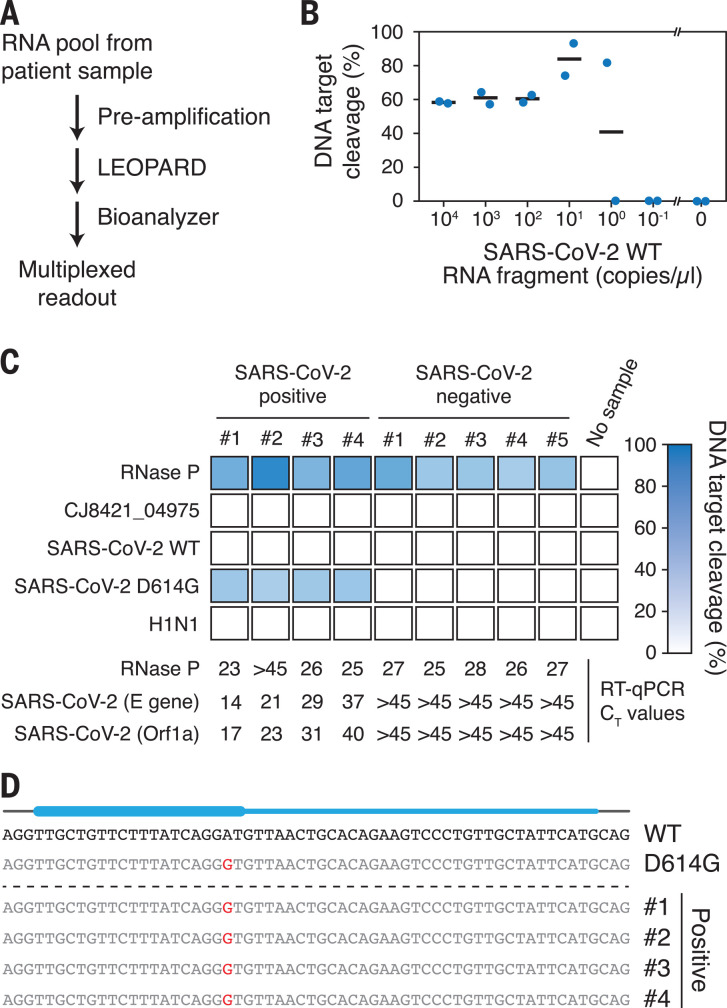
LEOPARD with RNA preamplification and Bioanalyzer readout allows for multiplexed detection of SARS-CoV-2 in patient samples. (**A**) General workflow for LEOPARD with target-specific preamplification and DNA target resolution on a Bioanalyzer. (**B**) Sensitivity of the workflow for detecting dilutions of an in vitro–transcribed WT SARS-CoV-2 RNA fragment. One microliter was added for each test. Bars represent the average of independent duplicates. (**C**) Multiplexed detection of five RNAs in patient samples confirmed positive or negative for SARS-CoV-2 by RT-qPCR. (**D**) Sanger sequencing results of the detected region in SARS-CoV-2 cDNA from the positive patient samples. Blue bar: Position of the ncrRNA, with the thick part indicating the resulting ncrRNA portion.

To extend LEOPARD beyond this proof-of-principle demonstration, we made two additions. First, we added target-specific reverse transcription–polymerase chain reaction (RT-PCR) and in vitro transcription similar to Cas13-based diagnostics (*29*) to improve assay sensitivity beyond the threshold set by detection of a cleaved DNA product. Second, we resolved DNA targets using a Bioanalyzer as a more practical readout (Fig. 5A). Applying this modified workflow to sense the in vitro–transcribed WT SARS-CoV-2 RNA fragment, we could detect as little as approximately one copy, or 1.7 aM in the original dilution, of this RNA (Fig. 5B and fig. S15A) compared with 3 × 10^8^ copies, or 0.6 nM in the original dilution, without preamplification (fig. S15B). As this sensitivity would be sufficient for detecting SARS-CoV-2 RNA in patient samples, we applied LEOPARD to evaluate samples confirmed positive or negative for SARS-CoV-2 by RT-qPCR (Fig. 5C and table S1). The positive samples reflected a range of SARS-CoV-2 RNA concentrations down to ~2 aM. We then probed for both SARS-CoV-2 and the D614G variant as well as influenza H1N1, the *C. jejuni* CJ8421_04975 mRNA (n4 under SpyCas9, Fig. 3D) as a nonhuman negative control, and the mRNA encoding human RNase P to confirm correct administration of the nasal swab using four Rptrs [one for both WT and D614G SARS-CoV-2 (Fig. 4E)] and five DNA targets. Of the four SARS-CoV-2–positive and five SARS-CoV-2–negative samples tested, RNase P mRNA but not the CJ8421_04975 mRNA or H1N1 RNA was detected in all nine samples (Fig. 5C and fig. S16). Notably, we detected the D614G variant of SARS-CoV-2 in all four positive samples, which was confirmed by sequencing preamplified cDNA (Fig. 5D). Although the sample size is small, detection of this variant suggests that it was spreading in Germany when the samples were collected. WT or D614G SARS-CoV-2 RNA was not detected in any of the negative samples, paralleling the RT-qPCR results (Fig. 5C). Each reaction allowed for parallel testing for five different RNAs, including controls, that would require separate reactions for other diagnostic platforms. These findings demonstrate the practical utility of LEOPARD for multiplexed RNA detection.

## Discussion

Starting from the characterization of a native CRISPR-Cas9 system in the bacterial pathogen *C. jejuni*, we discovered that cellular transcripts can be the source of noncanonical crRNAs through hybridization with the tracrRNA. This discovery adds ncrRNAs to the list of RNA guides found in nature, including crRNAs, scaRNAs that similarly pair with the tracrRNA anti-repeat, and “natural” sgRNAs formed through upstream transcription of the tracrRNA (*30*, *31*). These prior examples are all encoded within CRISPR-Cas loci. So far, it remains unclear whether ncrRNAs serve a physiological role in *C. jejuni*. Future studies therefore could help clarify whether ncrRNAs derived from outside CRISPR arrays are spurious off-target products that are tolerated by the host or if they confer yet-to-be-discovered functions extending beyond adaptive immunity.

We further demonstrated that Rptrs can link the presence of an RNA of interest to sequence-specific DNA targeting by Cas9. This capability could enable in vivo applications with Cas9 such as multiplexed transcriptional recording or transcription-dependent editing. The most immediate application involved multiplexed RNA detection in vitro through LEOPARD. LEOPARD adds to the existing CRISPR diagnostic platforms principally based on Cas12a or Cas13 (*24*–*26*, *29*) while offering scalable multiplexing in a single reaction. Our reliance on gel electrophoresis or a Bioanalyzer provided a proof-of-principle demonstration of multiplexed detection, although both are difficult to implement beyond a dozen targets. Instead, incorporating microarrays or next-generation sequencing (*32*) can potentially monitor up to millions of targets by linking the presence of a specific RNA to binding of labeled Cas9 or cleavage of labeled DNA target at a specific location on a chip. Either approach could also enhance assay sensitivity due to the limited number of DNA molecules in a given cluster, potentially circumventing the need for RNA preamplification. Simpler setups involving lateral flow assays could also be developed, paralleling other CRISPR diagnostics (*33*, *34*). With further development, LEOPARD could become a powerful diagnostic tool not only for the detection of viral variants distinguished by individual nts but also for applications such as screening for cancer mutations, identifying pathogens and antibiotic-resistance markers, or determining gene expression profiles for drug susceptibility. Moreover, extending Rptrs to tracrRNA-dependent nucleases within type V systems could help incorporate their distinctive attributes, such as signal amplification or programmable transposition (*24*, *35*).

While systematically perturbing the standard crRNA:tracrRNA duplex, we found that many deviations–particularly outside of the 5′ end of the repeat–were tolerated by Cas9 and still led to targeting of designed DNA targets. However, despite this promiscuity, targeting is still determined by the upstream guide sequence and the requirement for a flanking PAM. Thus, both anti-repeat hybridization and guide-dependent DNA targeting may limit off-targeting activity. Although we did not observe any detectable off-targeting in vitro or in vivo, future work could devise design rules for Rptrs that account for potential off-targeting as well as on-target activity, similar to existing sgRNA design algorithms (*36*). In turn, these rules would help advance the utilization of any RNA into a sequence-specific guide for CRISPR technologies.

## Supplementary Materials

science.sciencemag.org/content/372/6545/941/suppl/DC1 Materials and Methods Supplementary Text Figs. S1 to S16 Table S1 References (*39*–*52*) MDAR Reproducibility Checklist

## Appendix

View/request a protocol for this paper from *Bio-protocol*.

## References

[1] R. Barrangou, C. Fremaux, H. Deveau, M. Richards, P. Boyaval, S. Moineau, D. A. Romero, P. Horvath, CRISPR provides acquired resistance against viruses in prokaryotes. Science 315, 1709–1712 (2007). 10.1126/science.1138140 17379808

[2] K. S. Makarova, Y. I. Wolf, J. Iranzo, S. A. Shmakov, O. S. Alkhnbashi, S. J. J. Brouns, E. Charpentier, D. Cheng, D. H. Haft, P. Horvath, S. Moineau, F. J. M. Mojica, D. Scott, S. A. Shah, V. Siksnys, M. P. Terns, Č. Venclovas, M. F. White, A. F. Yakunin, W. Yan, F. Zhang, R. A. Garrett, R. Backofen, J. van der Oost, R. Barrangou, E. V. Koonin, Evolutionary classification of CRISPR-Cas systems: A burst of class 2 and derived variants. Nat. Rev. Microbiol. 18, 67–83 (2020). 10.1038/s41579-019-0299-x 31857715 PMC8905525

[3] S. J. J. Brouns, M. M. Jore, M. Lundgren, E. R. Westra, R. J. H. Slijkhuis, A. P. L. Snijders, M. J. Dickman, K. S. Makarova, E. V. Koonin, J. van der Oost, Small CRISPR RNAs guide antiviral defense in prokaryotes. Science 321, 960–964 (2008). 10.1126/science.1159689 18703739 PMC5898235

[4] G. J. Knott, J. A. Doudna, CRISPR-Cas guides the future of genetic engineering. Science 361, 866–869 (2018). 10.1126/science.aat5011 30166482 PMC6455913

[5] A. V. Anzalone, L. W. Koblan, D. R. Liu, Genome editing with CRISPR-Cas nucleases, base editors, transposases and prime editors. Nat. Biotechnol. 38, 824–844 (2020). 10.1038/s41587-020-0561-9 32572269

[6] E. Deltcheva, K. Chylinski, C. M. Sharma, K. Gonzales, Y. Chao, Z. A. Pirzada, M. R. Eckert, J. Vogel, E. Charpentier, CRISPR RNA maturation by trans-encoded small RNA and host factor RNase III. Nature 471, 602–607 (2011). 10.1038/nature09886 21455174 PMC3070239

[7] T. Karvelis, G. Gasiunas, A. Miksys, R. Barrangou, P. Horvath, V. Siksnys, crRNA and tracrRNA guide Cas9-mediated DNA interference in *Streptococcus thermophilus*. RNA Biol. 10, 841–851 (2013). 10.4161/rna.24203 23535272 PMC3737341

[8] M. Jinek, K. Chylinski, I. Fonfara, M. Hauer, J. A. Doudna, E. Charpentier, A programmable dual-RNA-guided DNA endonuclease in adaptive bacterial immunity. Science 337, 816–821 (2012). 10.1126/science.1225829 22745249 PMC6286148

[9] G. Dugar, R. T. Leenay, S. K. Eisenbart, T. Bischler, B. U. Aul, C. L. Beisel, C. M. Sharma, CRISPR RNA-dependent binding and cleavage of endogenous RNAs by the *Campylobacter jejuni* Cas9. Mol. Cell 69, 893–905.e7 (2018). 10.1016/j.molcel.2018.01.032 29499139 PMC5859949

[10] T. L. Bailey, M. Boden, F. A. Buske, M. Frith, C. E. Grant, L. Clementi, J. Ren, W. W. Li, W. S. Noble, MEME SUITE: Tools for motif discovery and searching. Nucleic Acids Res. 37, W202–W208 (2009). 10.1093/nar/gkp335 19458158 PMC2703892

[11] J. N. Zadeh, C. D. Steenberg, J. S. Bois, B. R. Wolfe, M. B. Pierce, A. R. Khan, R. M. Dirks, N. A. Pierce, NUPACK: Analysis and design of nucleic acid systems. J. Comput. Chem. 32, 170–173 (2011). 10.1002/jcc.21596 20645303

[12] R. T. Leenay, C. L. Beisel, Deciphering, communicating, and engineering the CRISPR PAM. J. Mol. Biol. 429, 177–191 (2017). 10.1016/j.jmb.2016.11.024 27916599 PMC5235977

[13] R. Marshall, C. S. Maxwell, S. P. Collins, T. Jacobsen, M. L. Luo, M. B. Begemann, B. N. Gray, E. January, A. Singer, Y. He, C. L. Beisel, V. Noireaux, Rapid and scalable characterization of CRISPR technologies using an *E. coli* cell-free transcription-translation system. Mol. Cell 69, 146–157.e3 (2018). 10.1016/j.molcel.2017.12.007 29304331 PMC5976856

[14] C. Liao, F. Ttofali, R. A. Slotkowski, S. R. Denny, T. D. Cecil, R. T. Leenay, A. J. Keung, C. L. Beisel, Modular one-pot assembly of CRISPR arrays enables library generation and reveals factors influencing crRNA biogenesis. Nat. Commun. 10, 2948 (2019). 10.1038/s41467-019-10747-3 31270316 PMC6610086

[15] T. Jacobsen, F. Ttofali, C. Liao, S. Manchalu, B. N. Gray, C. L. Beisel, Characterization of Cas12a nucleases reveals diverse PAM profiles between closely-related orthologs. Nucleic Acids Res. 48, 5624–5638 (2020). 10.1093/nar/gkaa272 32329776 PMC7261169

[16] M. Yamada, Y. Watanabe, J. S. Gootenberg, H. Hirano, F. A. Ran, T. Nakane, R. Ishitani, F. Zhang, H. Nishimasu, O. Nureki, Crystal structure of the minimal Cas9 from *Campylobacter jejuni* reveals the molecular diversity in the CRISPR-Cas9 systems. Mol. Cell 65, 1109–1121.e3 (2017). 10.1016/j.molcel.2017.02.007 28306506

[17] X. Wu, A. J. Kriz, P. A. Sharp, Target specificity of the CRISPR-Cas9 system. Quant. Biol. 2, 59–70 (2014). 10.1007/s40484-014-0030-x 25722925 PMC4338555

[18] H.-H. Wessels, A. Méndez-Mancilla, X. Guo, M. Legut, Z. Daniloski, N. E. Sanjana, Massively parallel Cas13 screens reveal principles for guide RNA design. Nat. Biotechnol. 38, 722–727 (2020). 10.1038/s41587-020-0456-9 32518401 PMC7294996

[19] T. Scott, R. Urak, C. Soemardy, K. V. Morris, Improved Cas9 activity by specific modifications of the tracrRNA. Sci. Rep. 9, 16104 (2019). 10.1038/s41598-019-52616-5 31695072 PMC6834579

[20] A. E. Briner, P. D. Donohoue, A. A. Gomaa, K. Selle, E. M. Slorach, C. H. Nye, R. E. Haurwitz, C. L. Beisel, A. P. May, R. Barrangou, Guide RNA functional modules direct Cas9 activity and orthogonality. Mol. Cell 56, 333–339 (2014). 10.1016/j.molcel.2014.09.019 25373540

[21] A. C. Reis, S. M. Halper, G. E. Vezeau, D. P. Cetnar, A. Hossain, P. R. Clauer, H. M. Salis, Simultaneous repression of multiple bacterial genes using nonrepetitive extra-long sgRNA arrays. Nat. Biotechnol. 37, 1294–1301 (2019). 10.1038/s41587-019-0286-9 31591552

[22] Y. Zhang, N. Heidrich, B. J. Ampattu, C. W. Gunderson, H. S. Seifert, C. Schoen, J. Vogel, E. J. Sontheimer, Processing-independent CRISPR RNAs limit natural transformation in *Neisseria meningitidis*. Mol. Cell 50, 488–503 (2013). 10.1016/j.molcel.2013.05.001 23706818 PMC3694421

[23] Y. Altuvia, A. Bar, N. Reiss, E. Karavani, L. Argaman, H. Margalit, *In vivo* cleavage rules and target repertoire of RNase III in *Escherichia coli*. Nucleic Acids Res. 46, 10530–10531 (2018). 10.1093/nar/gky816 30184218 PMC6212792

[24] J. S. Chen, E. Ma, L. B. Harrington, M. Da Costa, X. Tian, J. M. Palefsky, J. A. Doudna, CRISPR-Cas12a target binding unleashes indiscriminate single-stranded DNase activity. Science 360, 436–439 (2018). 10.1126/science.aar6245 29449511 PMC6628903

[25] A. East-Seletsky, M. R. O’Connell, S. C. Knight, D. Burstein, J. H. Cate, R. Tjian, J. A. Doudna, Two distinct RNase activities of CRISPR-C2c2 enable guide-RNA processing and RNA detection. Nature 538, 270–273 (2016). 10.1038/nature19802 27669025 PMC5576363

[26] J. S. Gootenberg, O. O. Abudayyeh, J. W. Lee, P. Essletzbichler, A. J. Dy, J. Joung, V. Verdine, N. Donghia, N. M. Daringer, C. A. Freije, C. Myhrvold, R. P. Bhattacharyya, J. Livny, A. Regev, E. V. Koonin, D. T. Hung, P. C. Sabeti, J. J. Collins, F. Zhang, Nucleic acid detection with CRISPR-Cas13a/C2c2. Science 356, 438–442 (2017). 10.1126/science.aam9321 28408723 PMC5526198

[27] B. Korber, W. M. Fischer, S. Gnanakaran, H. Yoon, J. Theiler, W. Abfalterer, N. Hengartner, E. E. Giorgi, T. Bhattacharya, B. Foley, K. M. Hastie, M. D. Parker, D. G. Partridge, C. M. Evans, T. M. Freeman, T. I. de Silva, C. McDanal, L. G. Perez, H. Tang, A. Moon-Walker, S. P. Whelan, C. C. LaBranche, E. O. Saphire, D. C. Montefiori, A. Angyal, R. L. Brown, L. Carrilero, L. R. Green, D. C. Groves, K. J. Johnson, A. J. Keeley, B. B. Lindsey, P. J. Parsons, M. Raza, S. Rowland-Jones, N. Smith, R. M. Tucker, D. Wang, M. D. Wyles; Sheffield COVID-19 Genomics Group, Tracking Changes in SARS-CoV-2 Spike: Evidence that D614G Increases Infectivity of the COVID-19 Virus. Cell 182, 812–827.e19 (2020). 10.1016/j.cell.2020.06.043 32697968 PMC7332439

[28] E. A. Boyle, J. O. L. Andreasson, L. M. Chircus, S. H. Sternberg, M. J. Wu, C. K. Guegler, J. A. Doudna, W. J. Greenleaf, High-throughput biochemical profiling reveals sequence determinants of dCas9 off-target binding and unbinding. Proc. Natl. Acad. Sci. U.S.A. 114, 5461–5466 (2017). 10.1073/pnas.1700557114 28495970 PMC5448226

[29] C. M. Ackerman, C. Myhrvold, S. G. Thakku, C. A. Freije, H. C. Metsky, D. K. Yang, S. H. Ye, C. K. Boehm, T. F. Kosoko-Thoroddsen, J. Kehe, T. G. Nguyen, A. Carter, A. Kulesa, J. R. Barnes, V. G. Dugan, D. T. Hung, P. C. Blainey, P. C. Sabeti, Massively multiplexed nucleic acid detection with Cas13. Nature 582, 277–282 (2020). 10.1038/s41586-020-2279-8 32349121 PMC7332423

[30] R. E. Workman, T. Pammi, B. T. K. Nguyen, L. W. Graeff, E. Smith, S. M. Sebald, M. J. Stoltzfus, C. W. Euler, J. W. Modell, A natural single-guide RNA repurposes Cas9 to autoregulate CRISPR-Cas expression. Cell 184, 675–688.e19 (2021). 10.1016/j.cell.2020.12.017 33421369

[31] H. K. Ratner, A. Escalera-Maurer, A. Le Rhun, S. Jaggavarapu, J. E. Wozniak, E. K. Crispell, E. Charpentier, D. S. Weiss, Catalytically active Cas9 mediates transcriptional interference to facilitate bacterial virulence. Mol. Cell 75, 498–510.e5 (2019). 10.1016/j.molcel.2019.05.029 31256988 PMC7205310

[32] C. Jung, J. A. Hawkins, S. K. Jones Jr.., Y. Xiao, J. R. Rybarski, K. E. Dillard, J. Hussmann, F. A. Saifuddin, C. A. Savran, A. D. Ellington, A. Ke, W. H. Press, I. J. Finkelstein, Massively parallel biophysical analysis of CRISPR-Cas complexes on next generation sequencing chips. Cell 170, 35–47.e13 (2017). 10.1016/j.cell.2017.05.044 28666121 PMC5552236

[33] J. P. Broughton, X. Deng, G. Yu, C. L. Fasching, V. Servellita, J. Singh, X. Miao, J. A. Streithorst, A. Granados, A. Sotomayor-Gonzalez, K. Zorn, A. Gopez, E. Hsu, W. Gu, S. Miller, C.-Y. Pan, H. Guevara, D. A. Wadford, J. S. Chen, C. Y. Chiu, CRISPR-Cas12-based detection of SARS-CoV-2. Nat. Biotechnol. 38, 870–874 (2020). 10.1038/s41587-020-0513-4 32300245 PMC9107629

[34] J. S. Gootenberg, O. O. Abudayyeh, M. J. Kellner, J. Joung, J. J. Collins, F. Zhang, Multiplexed and portable nucleic acid detection platform with Cas13, Cas12a, and Csm6. Science 360, 439–444 (2018). 10.1126/science.aaq0179 29449508 PMC5961727

[35] J. Strecker, A. Ladha, Z. Gardner, J. L. Schmid-Burgk, K. S. Makarova, E. V. Koonin, F. Zhang, RNA-guided DNA insertion with CRISPR-associated transposases. Science 365, 48–53 (2019). 10.1126/science.aax9181 31171706 PMC6659118

[36] R. E. Hanna, J. G. Doench, Design and analysis of CRISPR-Cas experiments. Nat. Biotechnol. 38, 813–823 (2020). 10.1038/s41587-020-0490-7 32284587

[37] T. Bischler, K. U. Förstner, D. Maticzka, P. R. Wright, PEAKachu: A peak calling tool for CLIP/RIP-seq data, Version 0.2.0, Zenodo (2021). https://zenodo.org/record/4669966.

[38] L. Barquist, Perl script for evaluating tracr:target interactions with Nupack, Zenodo (2021). https://zenodo.org/record/4668694.

[39] W. Jiang, D. Bikard, D. Cox, F. Zhang, L. A. Marraffini, RNA-guided editing of bacterial genomes using CRISPR-Cas systems. Nat. Biotechnol. 31, 233–239 (2013). 10.1038/nbt.2508 23360965 PMC3748948

[40] E. Kim, T. Koo, S. W. Park, D. Kim, K. Kim, H.-Y. Cho, D. W. Song, K. J. Lee, M. H. Jung, S. Kim, J. H. Kim, J. H. Kim, J.-S. Kim, *In vivo* genome editing with a small Cas9 orthologue derived from *Campylobacter jejuni*. Nat. Commun. 8, 14500 (2017). 10.1038/ncomms14500 28220790 PMC5473640

[R41] 41. P. Horvath, D. A. Romero, A.-C. Coûté-Monvoisin, M. Richards, H. Deveau, S. Moineau, P. Boyaval, C. Fremaux, R. Barrangou, Diversity, activity, and evolution of CRISPR loci in *Streptococcus thermophilus*. J. Bacteriol. 190, 1401–1412 (2008). 10.1128/JB.01415-07 18065539 PMC2238196

[R42] 42. K. M. Esvelt, P. Mali, J. L. Braff, M. Moosburner, S. J. Yaung, G. M. Church, Orthogonal Cas9 proteins for RNA-guided gene regulation and editing. Nat. Methods 10, 1116–1121 (2013). 10.1038/nmeth.2681 24076762 PMC3844869

[43] R. T. Leenay, K. R. Maksimchuk, R. A. Slotkowski, R. N. Agrawal, A. A. Gomaa, A. E. Briner, R. Barrangou, C. L. Beisel, Identifying and visualizing functional PAM diversity across CRISPR-Cas systems. Mol. Cell 62, 137–147 (2016). 10.1016/j.molcel.2016.02.031 27041224 PMC4826307

[44] G. Dugar, A. Herbig, K. U. Förstner, N. Heidrich, R. Reinhardt, K. Nieselt, C. M. Sharma, High-resolution transcriptome maps reveal strain-specific regulatory features of multiple *Campylobacter jejuni* isolates. PLOS Genet. 9, e1003495 (2013). 10.1371/journal.pgen.1003495 23696746 PMC3656092

[45] G. Dugar, S. L. Svensson, T. Bischler, S. Wäldchen, R. Reinhardt, M. Sauer, C. M. Sharma, The CsrA-FliW network controls polar localization of the dual-function flagellin mRNA in *Campylobacter jejuni*. Nat. Commun. 7, 11667 (2016). 10.1038/ncomms11667 27229370 PMC4894983

[46] M. Martin, Cutadapt removes adapter sequences from high-throughput sequencing reads. EMBnet.journal 17, 10–12 (2011). 10.14806/ej.17.1.200

[47] K. U. Förstner, J. Vogel, C. M. Sharma, READemption-a tool for the computational analysis of deep-sequencing-based transcriptome data. Bioinformatics 30, 3421–3423 (2014). 10.1093/bioinformatics/btu533 25123900

[48] S. Hoffmann, C. Otto, S. Kurtz, C. M. Sharma, P. Khaitovich, J. Vogel, P. F. Stadler, J. Hackermüller, Fast mapping of short sequences with mismatches, insertions and deletions using index structures. PLOS Comput. Biol. 5, e1000502 (2009). 10.1371/journal.pcbi.1000502 19750212 PMC2730575

[49] M. I. Love, W. Huber, S. Anders, Moderated estimation of fold change and dispersion for RNA-seq data with DESeq2. Genome Biol. 15, 550 (2014). 10.1186/s13059-014-0550-8 25516281 PMC4302049

[50] J. Shin, V. Noireaux, Efficient cell-free expression with the endogenous *E. coli* RNA polymerase and σ factor 70. J. Biol. Eng. 4, 8 (2010). 10.1186/1754-1611-4-8 20576148 PMC3161345

[51] T. Afroz, K. Biliouris, Y. Kaznessis, C. L. Beisel, Bacterial sugar utilization gives rise to distinct single-cell behaviours. Mol. Microbiol. 93, 1093–1103 (2014). 10.1111/mmi.12695 24976172 PMC4160389

[52] K. B. Hummel, L. Lowe, W. J. Bellini, P. A. Rota, Development of quantitative gene-specific real-time RT-PCR assays for the detection of measles virus in clinical specimens. J. Virol. Methods 132, 166–173 (2006). 10.1016/j.jviromet.2005.10.006 16274752

